# Supplementary material for: Dynamics of bacterial and archaeal communities during horse bedding and green waste composting
Source: PeerJ. 2023 May 3;11:e15239. doi: 10.7717/peerj.15239 (PMC10163874; doi:10.7717/peerj.15239)

Differently abundant putative species involved in cellulose degradation

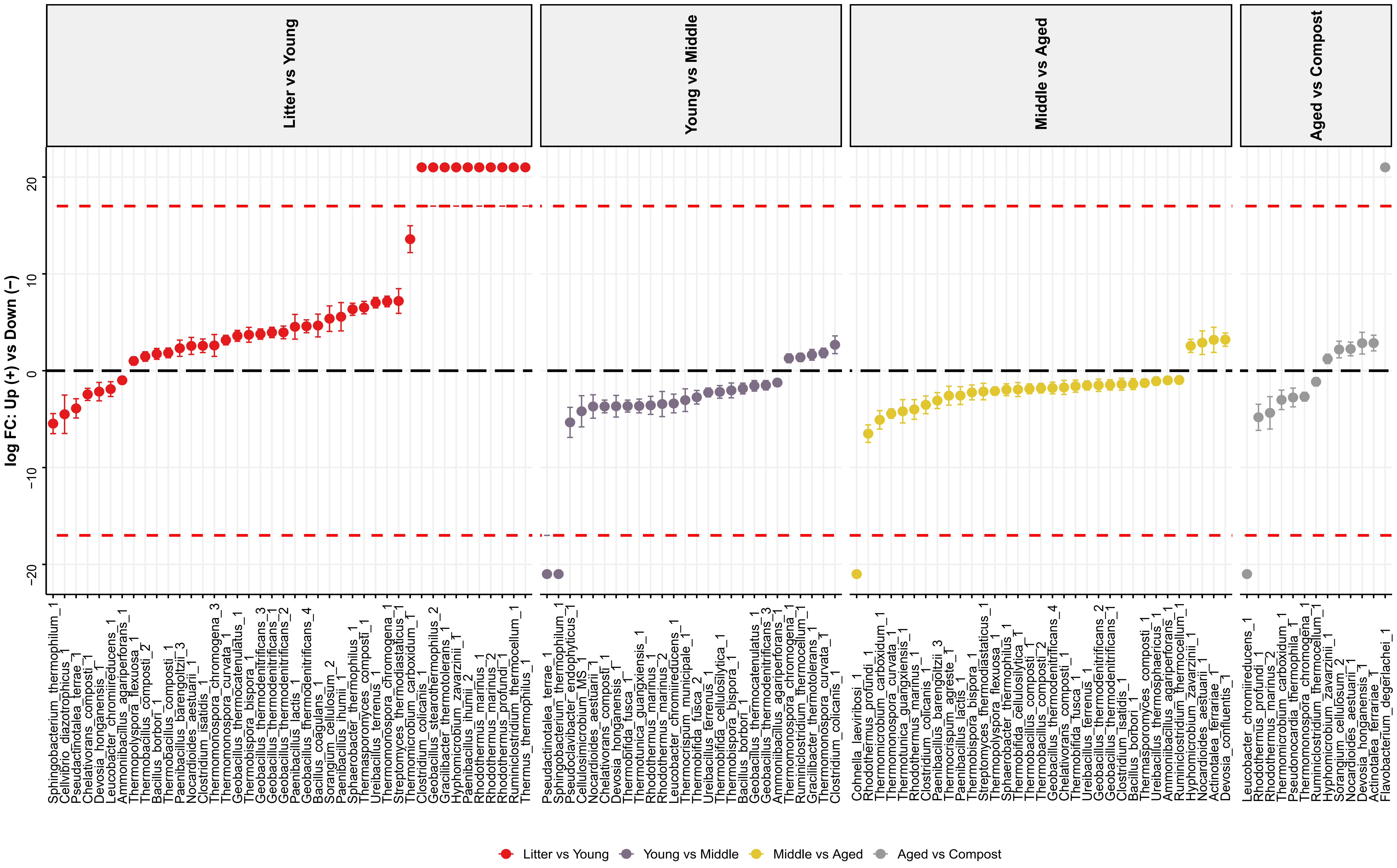

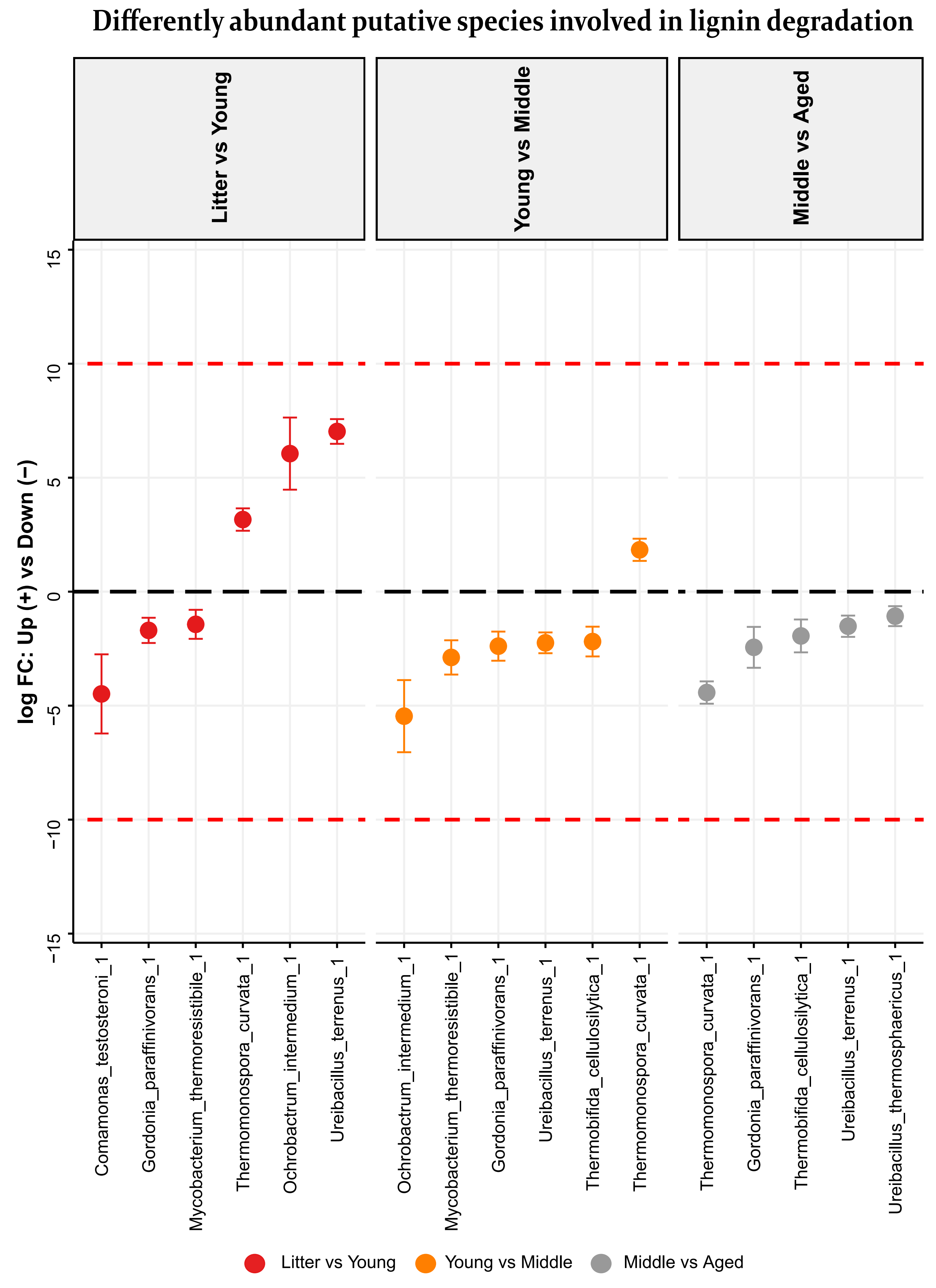
Differently abundant putative species involved in lignin degradation
Litter vs Young
Young vs Middle
Middle vs Aged
log FC: Up (+) vs Down (−)
15
10
5
0
−5
−10
−15
Comamonas_testosteroni_1
Gordonia_paraffinivorans_1
Mycobacterium_thermoresistibile_1
Thermomonospora_curvata_1
Ochrobactrum_intermedium_1
Ureibacillus_terrenus_1
Ochrobactrum_intermedium_1
Mycobacterium_thermoresistibile_1
Gordonia_paraffinivorans_1
Ureibacillus_terrenus_1
Thermobifida_cellulosilytica_1
Thermomonospora_curvata_1
Thermomonospora_curvata_1
Gordonia_paraffinivorans_1
Thermobifida_cellulosilytica_1
Ureibacillus_terrenus_1
Ureibacillus_thermosphaericus_1
Litter vs Young
Young vs Middle
Middle vs Aged

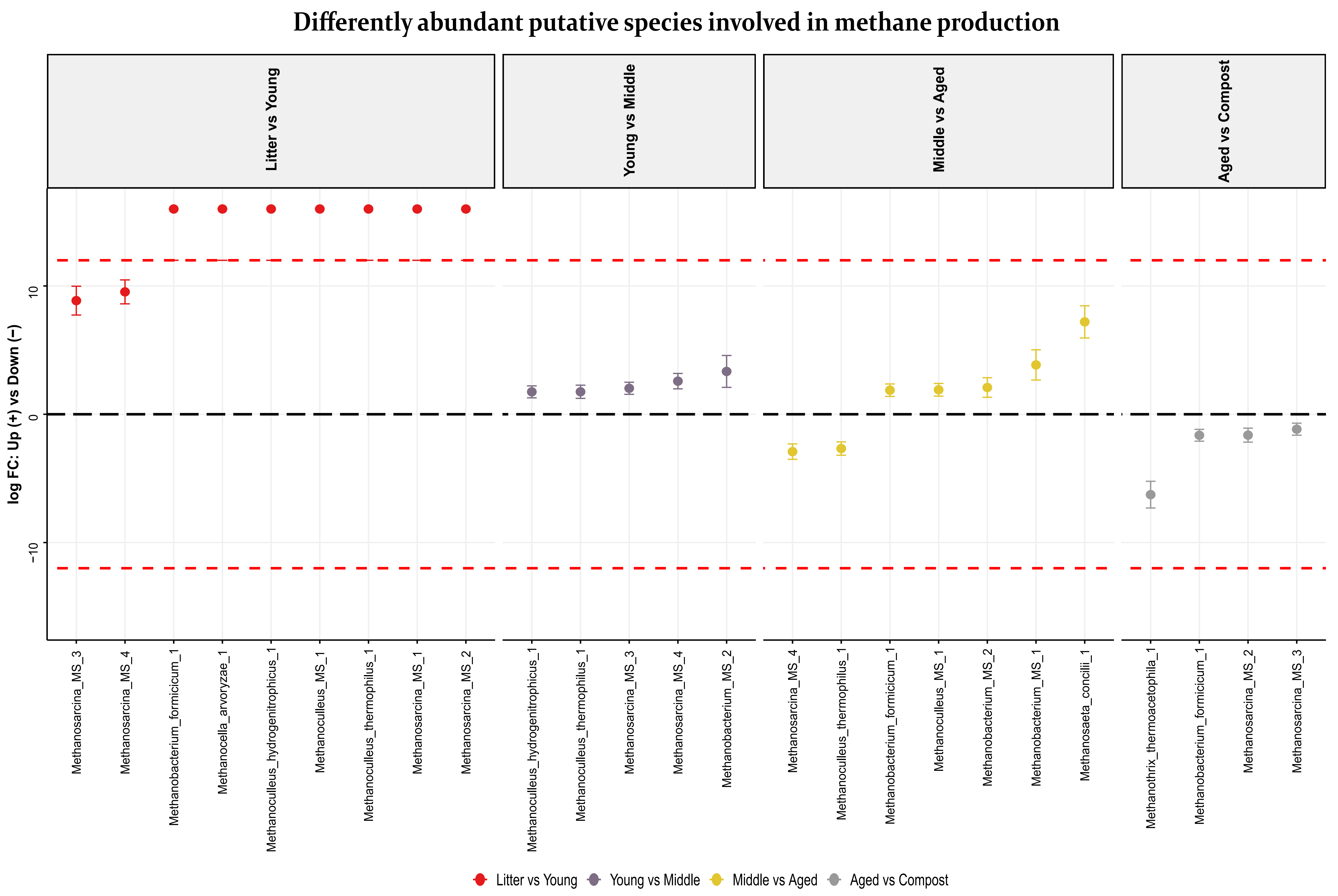

Differently abundant putative species involved in methane production
Litter vs Young
Young vs Middle
Middle vs Aged
Aged vs Compost
log FC: Up (+) vs Down (−)
10
0
−10
Methanosarcina_MS_3
Methanosarcina_MS_4
Methanobacterium_formicicum_1
Methanocella_arvoryzae_1
Methanoculleus_hydrogenitrophicus_1
Methanoculleus_MS_1
Methanoculleus_thermophilus_1
Methanosarcina_MS_1
Methanosarcina_MS_2
Methanoculleus_hydrogenitrophicus_1
Methanoculleus_thermophilus_1
Methanosarcina_MS_3
Methanosarcina_MS_4
Methanobacterium_MS_2
Methanosarcina_MS_4
Methanoculleus_thermophilus_1
Methanobacterium_formicicum_1
Methanoculleus_MS_1
Methanobacterium_MS_2
Methanobacterium_MS_1
Methanosaeta_concilii_1
Methanothrix_thermoacetophila_1
Methanobacterium_formicicum_1
Methanosarcina_MS_2
Methanosarcina_MS_3
Litter vs Young
Young vs Middle
Middle vs Aged
Aged vs Compost

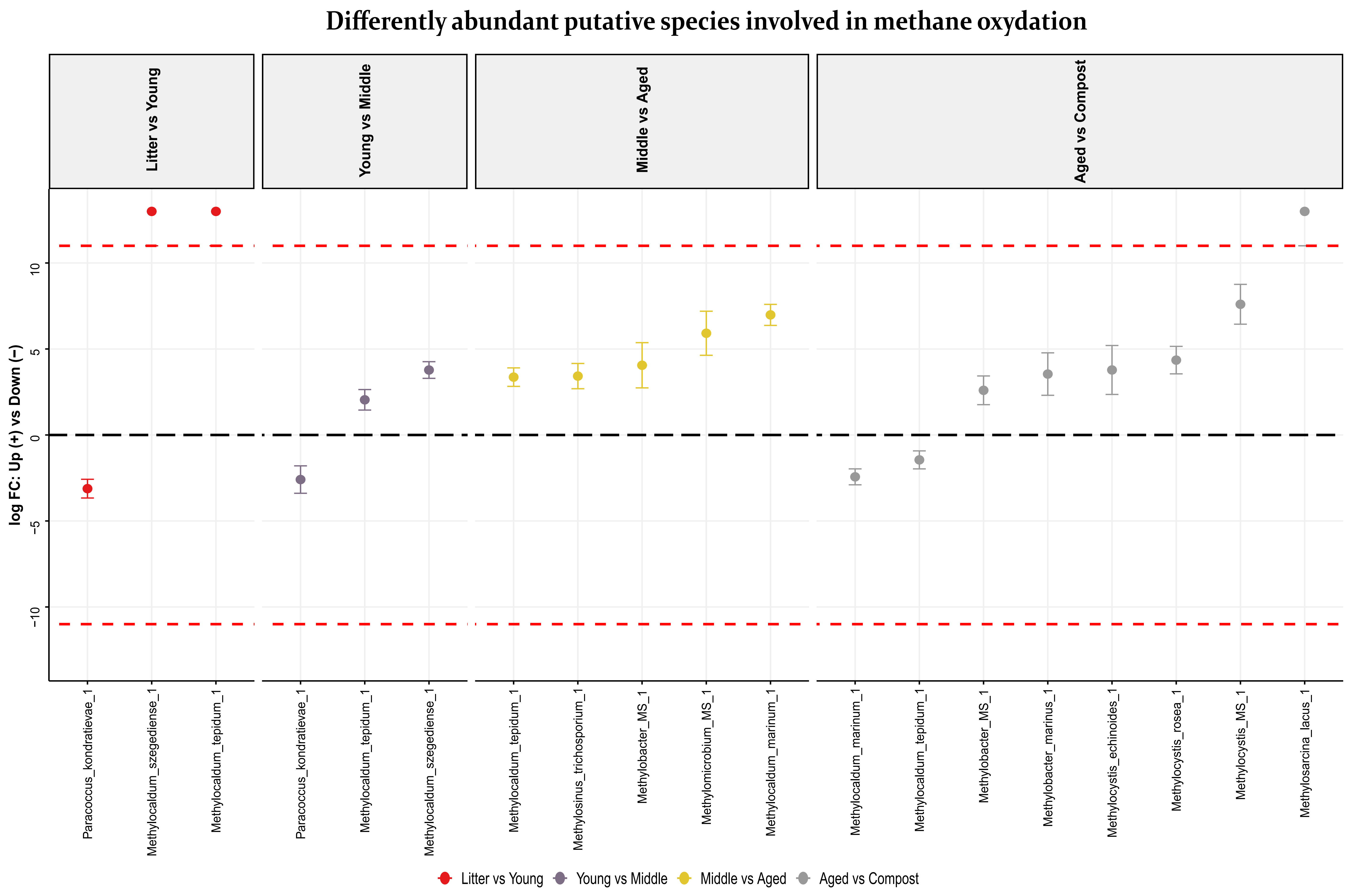

Differently abundant putative species involved in methane oxydation
Litter vs Young
Young vs Middle
Middle vs Aged
Aged vs Compost
log FC: Up (+) vs Down (−)
10
5
0
−5
−10
Paracoccus_kondratievae_1
Methylocaldum_szegediense_1
Methylocaldum_tepidum_1
Paracoccus_kondratievae_1
Methylocaldum_tepidum_1
Methylocaldum_szegediense_1
Methylocaldum_tepidum_1
Methylosinus_trichosporium_1
Methylobacter_MS_1
Methylomicrobium_MS_1
Methylocaldum_marinum_1
Methylocaldum_marinum_1
Methylocaldum_tepidum_1
Methylobacter_MS_1
Methylobacter_marinus_1
Methylocystis_echinoides_1
Methylocystis_rosea_1
Methylocystis_MS_1
Methylosarcina_lacus_1
Litter vs Young
Young vs Middle
Middle vs Aged
Aged vs Compost

Differently abundant putative species involved in nitrogen cycle

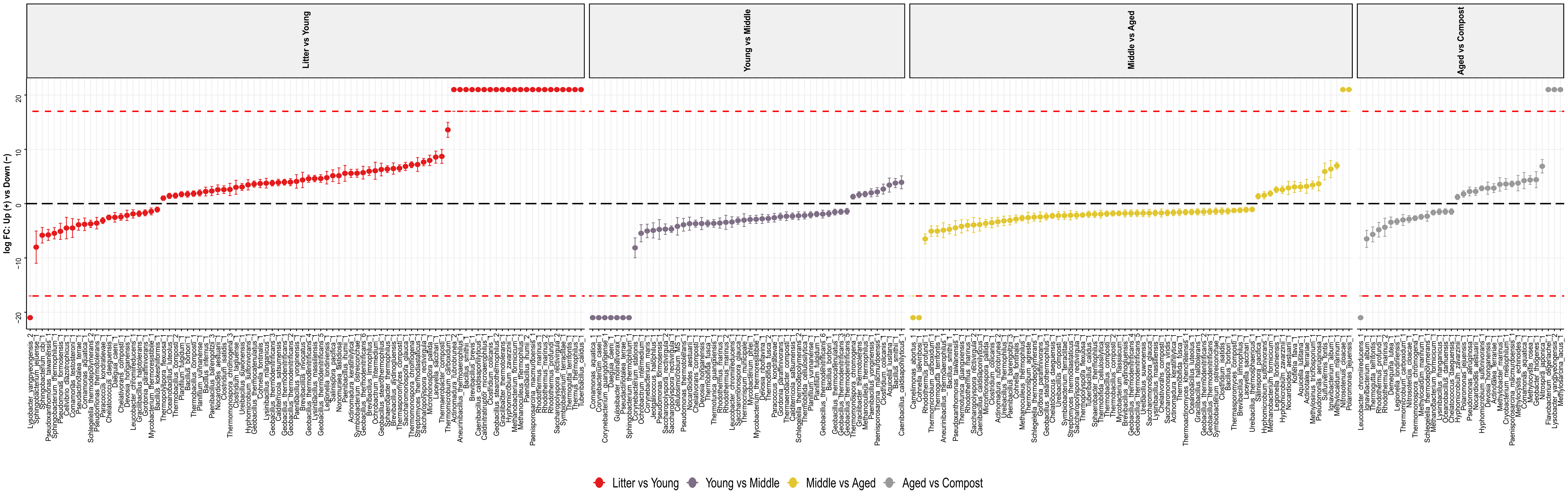

Supplement: Supplemental Information 3 — Fold change (FC log2) denotes relative differences in relative abundance between successive sampled phase (DESeq2); Litter vs. Young, Young vs. Middle, Middle vs. Aged and Aged vs. Compost [file peerj-11-15239-s003.pdf]
